# Dopamine–prolactin pathway potentially contributes to the schizophrenia and type 2 diabetes comorbidity

**DOI:** 10.1038/tp.2016.50

**Published:** 2016-04-19

**Authors:** C Gragnoli, G M Reeves, J Reazer, T T Postolache

**Affiliations:** 1 Division of Endocrinology, Diabetes, and Metabolism, Department of Medicine, University of Florida College of Medicine, Jacksonville, FL, USA; 2 Department of Public Health Sciences, Penn State College of Medicine, Hershey, PA, USA; 3 Molecular Biology Laboratory, Bios Biotech Multi-Diagnostic Health Center, Rome, Italy; 4 Division of Child and Adolescent Psychiatry, Department of Psychiatry, University of Maryland School of Medicine, Baltimore, MD, USA; 5 Borland Health Sciences Library, University of Florida, Jacksonville, FL, USA; 6 Rocky Mountain Mental Illness Research Education and Clinical Center, Denver, CO, USA; 7 Veterans Integrated Service Network 5 MIRECC, Baltimore, MD, USA; 8 Department of Psychiatry, University of Maryland, Baltimore, MD, USA

## Abstract

Schizophrenia (SCZ) and type 2 diabetes (T2D) are clinically associated, and common knowledge attributes this association to side effects of antipsychotic treatment. However, even drug-naive patients with SCZ are at increased risk for T2D. Dopamine dysfunction has a central role in SCZ. It is well-known that dopamine constitutively inhibits prolactin (PRL) secretion via the dopamine receptor 2 (DR2D). If dopamine is increased or if dopamine receptors hyperfunction, PRL may be reduced. During the first SCZ episode, low PRL levels are associated with worse symptoms. PRL is essential in human and social bonding, as well as it is implicated in glucose homeostasis. Dopamine dysfunction, beyond contributing to SCZ symptoms, may lead to altered appetite and T2D. To our knowledge, there are no studies of the genetics of the SCZ–T2D comorbidity focusing jointly on the dopamine and PRL pathway in the attempt to capture molecular heterogeneity correlated to possible disease manifestation heterogeneity. In this dopamine–PRL pathway-focused-hypothesis-driven review on the association of SCZ with T2D, we report a specific revision of what it is known about PRL and dopamine in relation to what we theorize is one of the missing links between the two disorders. We suggest that new studies are necessary to establish the genetic role of PRL and dopamine pathway in SCZ–T2D comorbidity.

## Introduction

Type 2 diabetes (T2D) is a polygenic complex disorder with a higher incidence of polygenic psychiatric diseases, including schizophrenia (SCZ).^1, 2^ Genetic factors of mental complex disorders may be common for some diseases.^3, 4, 5^ Even though antidepressants and antipsychotics may cause metabolic dysfunction, some psychiatric disorders increase T2D risk, independently of the therapy.^6, 7^ Given that T2D is a leading cause of morbidity and mortality among individuals with SCZ, there is a compelling need to identify mechanisms that increase vulnerability for T2D among individuals with psychotic illness.^8^ In this review, we only focus on a pre-treatment SCZ model as an increased risk factor for T2D; we will not discuss the well-known effects of antipsychotics on weight gain or T2D. Of interest, Kirkpatrick *et al.*^9^ support re-conceptualization of SCZ as a systemic disorder, rather than simply a brain disease. In this paper, we outline evidence to support this theory, as well as highlight genetic research needs to study the dopamine and prolactin (PRL) pathway to investigate possible underlying etiopathogenesis linking psychiatric and metabolic impairment, more specifically the comorbidity of SCZ and T2D. Our hypothesis is that neuroendocrine dysfunction in SCZ confers risk for T2D and that shared risk factors contribute to the clinical SCZ–T2D association.

## T2D and SCZ

T2D has 8.3% prevalence worldwide^10^ and causes significant morbidity and mortality.^11, 12^ T2D onset is usually after 40 years of age, but with increasing incidence at younger ages.^13^

SCZ, a severe chronic illness with 1% prevalence, is characterized by positive or negative symptoms for a 1-month period, with disturbance signs ⩾6 months, in addition to continuous social dysfunction. The individual must have at least one of these three positive symptoms: delusions (mostly persecutory); hallucinations (visual or auditory); and disorganized speech; negative symptoms include flat affect and lack of purposeful action.^14^ Disease onset is commonly between ages 15 and 25.^15^

SCZ and T2D are both heterogeneous complex disorders, considered by most to be determined by polygenic causes, thus by several genes with variations of modest effect. Several pathways, including dopaminergic, serotonergic, glutamatergic, cholinergic and GABAergic, have been implicated in SCZ.^16, 17^

### Evidence of the SCZ–T2D association

The epidemiology of T2D among individuals with psychotic illness was recently reviewed by Ward and Druss:^18^ T2D prevalence ranges from 1–26% to 50% across studies of individuals with psychotic disorders, with median prevalence of 13%. Clinical evidence of the association of SCZ and T2D derives from several studies. Drug-naive patients with first-episode SCZ have increased prevalence of impaired fasting glucose tolerance and are more insulin resistant with higher glycemia and insulinemia compared with control subjects.^19^ Patients are matched with control subjects for age, gender, lifestyle and anthropometric measures; however, they also have higher cortisol levels than the healthy subjects.^19^ In another study, antipsychotic-naive subjects with non-affective psychosis have a significant increased prevalence of impaired glucose tolerance compared with the control group (psychosis group 16% versus control group 0%), which is not due to differences in age, gender, ethnicity, neighborhood of residence, socioeconomic status, body mass index, smoking, aerobic conditioning or cortisolemia.^20^ The study strength is due to the consideration of several potential confounders, including cortisol levels.

Moreover, drug-naive patients with SCZ/schizoaffective disorder, compared with matched healthy subjects, have hepatic insulin resistance (a T2D trait), not attributable to visceral fat mass differences or factors associated with hepatic insulin resistance, indicating a link between SCZ and hepatic insulin resistance.^21^ The study is well conducted as it excludes patients with the following: antipsychotics/any other medication use except for acetaminophen; diabetes; medical/family T2D history; recent history of alcohol abuse or alcohol/cannabis use; alcohol/psychoactive substance abuse dependence disorder; and any somatic illness (for example, metabolic/endocrine diseases, active infection or brain gross structural abnormalities on magnetic resonance imaging). The healthy subjects are matched for age, gender and body mass index.^21^

In addition, glucose metabolites are differentially expressed in peripheral blood mononuclear cells of drug-naive first-episode SCZ subjects versus healthy controls: seven metabolites are significantly increased (glucose, glucose 6-phosphate, fructose, fructose 6-phosphate, glycerate 3-phosphate, succinic acid and ribose 5-phosphate) and four metabolites are significantly decreased (glyceraldehyde-3-phosphate, dihydroxyacetone phosphate, glycerol 3-phosphate and citric acid).^22^ The limitations of this study are the sample size and lack of ethnic diversity.^22^

Of interest, drug-naive SCZ patients and their first-degree relatives have increased prevalence of impaired glucose tolerance compared with healthy controls.^23^ Patients and control subjects are matched for age, gender, ethnicity, smoking and alcohol intake.^23^ Another study show that siblings of SCZ probands have a significantly increased impaired glucose tolerance compared with the control subjects.^24^ The study subjects are matched for age, gender, neighborhood of residence, socio-economic status, body mass index and smoking, but the sample size is small. Newly diagnosed non-affective psychosis and control subjects are interviewed for parental history of T2D, and psychosis is found to be a significant predictor of T2D in either parent. This study is robust as it is performed using a logistic regression model, including multiple potential confounders. The authors conclude that the increased T2D prevalence in the parents of non-affective psychosis subjects may be caused by shared genetic or environmental risk factors, or both.^25^

All above-mentioned studies are not large-scale studies due to the fact the even large specialized medical centers are able to recruit each year only 10–20 patients who are antipsychotic-naive, and free of substance abuse and comorbidities. To summarize, these studies show that drug-naive SCZ patients have impaired glucose levels, insulin action and increased T2D risk.

### Clinical studies of PRL, inflammation, cardiovascular mortality and metabolism

A large population-based Study of Health in Pomerania reveals that PRL levels are significantly associated with the inflammatory biomarker interleukin-6 and white blood cells counts, indicating a possible role of PRL in inflammation.^26^ This is of relevance considering the role of inflammation in T2D.^27^ After adjusting for multiple variables, Haring *et al.* identify in a very large population-based Study of Health in Pomerania a significant association of continuous PRL with all-cause mortality and cardiovascular death-specific mortality in men and women. They find that subjects with the highest PRL tertile have the highest mortality risk compared with subjects with the lowest PRL tertile.^28^ In the same population, Balbach *et al.*^29^ show, after multivariable adjustment, an inverse association of low PRL and T2D risk but not of metabolic syndrome risk. Even though the authors use a large sample and longitudinal analyses do not support an association of PRL with metabolic syndrome or T2D, they only test Caucasians and a single non-fasting PRL level, which does not reflect PRL pulsatility. Also, they do not rule out endocrine disorders causing T2D (for example, hyperthyroidism, acromegaly and Cushing syndrome).^29^

### SCZ subtypes and glucose metabolism

SCZ phenotypes are differently associated with T2D: non-deficit-SCZ patients (who have more positive symptoms such as hallucinations rather than negative symptoms such as anhedonia) have higher glucose levels than deficit-SCZ patients (who have more negative symptoms rather than positive symptoms), and that the latter have higher glucose levels than the control subjects. Thus, each SCZ phenotype, characterized mostly by persistent positive symptoms versus mainly persistent negative symptoms, may entail a distinct glucose impairment etiology.^30^ As deficit-SCZ patients tend to be less active physically,^31^ their sedentary lifestyle may not explain their reduced, rather than increased, glucose levels compared with the non-deficit-SCZ patients. Interestingly, a study in SCZ subjects shows an association of anhedonia, a feature of deficit-SCZ, with subtle differences in food liking, possibly based on a lower hedonic threshold for food.^32^ Hence, it is very likely that deficit-SCZ subjects tend to eat less as they derive less pleasure from the act of eating. However, other factors will need to explain the lower glucose levels compared with the non-deficit-SCZ subjects.

## Dopamine and PRL data in SCZ and T2D

We believe that there are common gene pathways responsible for the SCZ–T2D association in drug-naive subjects. In particular, we propose that the *PRL* and dopamine gene pathways may contribute to both T2D and SCZ, and/or to any metabolic and psychological associated trait predisposing to both diseases.

### Dopaminergic and PRL pathway in SCZ

Our hypothesis stems from the evidence that SCZ etiology is caused in part by the activation of dopamine receptor 2 (DR2D), due to increased dopamine levels and increased DR2D receptor density in the striatum, and by decreased dopaminergic activity in the prefrontal cortex.^33^ Therefore, the activation of DR2D receptors in non-cortical areas and function reduction of dopamine or dopamine receptors in cortical areas contribute to SCZ. Increased dopamine activity is also implicated in impaired reinforcement learning in SCZ.^34^ In addition, research on antipsychotic mechanisms of action has primarily focused on therapeutic effects on dopamine and related systems.^35^

It is important to note that dopamine is the PRL-inhibitory factor, and PRL levels appear to be both decreased in drug-free female patients with SCZ in some studies,^36^ as well as increased in drug-naive patients in other studies, with exclusion of differences attributable to age, gender, smoking, body mass index, ethnicity or socioeconomic status.^37^

PRL levels are higher in females than in males and vary in females across menstrual cycle. Of interest, male and female differences in age of onset, course and prognosis of SCZ suggest possible gender differences in underlying molecular mechanisms of disease pathogenesis. In fact, males have higher SCZ prevalence, earlier age of onset and poorer clinical prognosis.^38^ Gene–sex interactions have been identified for enzymes that catalyze dopamine metabolism.^38^

While Jose *et al.*^39^ report that PRL levels are higher in drug-free SCZ subjects than in healthy individuals and are significantly associated with severity of psychopathology, suicide risk and negative symptoms, other studies suggest possible gender discrepancies on the PRL association with different psychological symptoms. Ramsey *et al.*^40^ show that PRL level is negatively associated with positive psychosis symptom scores in male drug-naive SCZ subjects; however, the female menstrual cycle is not reported. First-episode drug-naive SCZ male patients have higher levels of PRL compared with control subjects and that the worsening of psychopathologic symptoms presents with PRL level reduction in both females and males.^41^ In a study, morning serum PRL levels are assessed among males who are either (1) drug-naive first-episode psychosis patients, (2) drug-free individuals with SCZ or (3) healthy controls: PRL levels are highest among the drug-naive first-episode patients, and both clinical groups had higher PRL levels compared with the healthy control group.^42^

Of interest, in drug-naive SCZ patients the PRL response to d-fenfluramine, an agent enhancing PRL release, is increased compared with control subjects, closely matched by age, weight, menstrual phase, gender and race.^43^ As fenfluramine increases serotonin, which increases PRL, this finding suggests either possible increased serotonergic tone or increased PRL response to serotonin in drug-naive SCZ patients.

The correlation of higher PRL levels and drug-naive SCZ subjects may be due to PRL receptor (PRLR) resistance, which may lead to reduced PRL action in brain areas important for social and cognitive functions.

We recognize that PRL increases during stress and has a role in social interaction and personal bonding,^44^ as well as in dissociative symptoms of depression.^45^ PRL is relevant to the mental adequacy to cope with social and personal stress,^46^ a major area of impairment in SCZ. We know that early stressful life events increase the risk of developing SCZ;^47^ thus, individuals genetically at risk for having inadequate social skills may, if exposed early in life to strong environmental and family stressors, such as parental abuse, develop SCZ.^47^ In a study, elevated PRL level and ‘burnout', a measure of psychological stress, are directly associated in males only; however, females are tested in the same phase of menstrual cycle.^48^

### Dopaminergic and PRL pathway in T2D

There is evidence from the literature of dopaminergic pathway and PRL involvement in metabolism. Dopamine synthesis and DR3D receptor activation in pancreatic beta cells regulates insulin secretion and intracellular calcium oscillations, which are related to insulin secretion.^49^ DR2D dopamine receptor-increased activity reduces food intake,^50^ explaining in part why a subject with T2D would benefit from bromocriptine, a dopamine agonist acting on DR2D.^51^ Also, DR2D dopamine receptor-increased activity decreases PRL levels, which in a non-diabetic young subject is associated with insulin resistance, whereas in an older T2D patient it is related to improved insulin sensitivity,^52^ the latter being another possible positive effect of bromocriptine in established T2D. Accordingly, a young individual with a constitutionally increased activity of DR2D and consequential lower PRL levels may be at increased risk for SCZ and T2D. In fact, PRL is essential for pancreatic and beta-cell embryogenesis and ontogenesis, including the perinatal period critical for establishing functional beta-cell reserve and adult insulin secretion capability. Indeed, constitutively decreased PRL levels may impair beta-cell mass growth,^53, 54^ thus increasing T2D risk. Further, PRL inhibits beta-cell apoptosis,^55, 56^ increases beta-cell survival^57^ and has a role in glucose regulation via glucokinase activity and in insulin secretion.^58, 59^ Moreover, PRLR is essential for insulin-producing-cell survival^60^ and islets adaptation in pregnancy.^61^ PRL via the PRLR has a role in beta-cell embryogenesis and ontogenesis, glucose homeostasis and beta-cell mass preservation during the insulin-resistant state of pregnancy.^62^

In addition, dopamine elevation and altered dopamine pathway function in SCZ may reduce PRL, thus while impairing glucose homeostasis,^58^ independently alter appetite,^63, 64^ induce carbohydrate craving^65^ and contribute to obesity or to T2D onset.^66, 67^

### Related gene studies in SCZ and T2D

Association studies focusing only on selected common gene variants of *DR1D*,^68^ *DR2D*,^69^ *DR3D*,^70^ *DR4D*^71^ and *DR5D*,^72^ and linkage studies^73^ show inconsistent results in SCZ. One study reports a positive association of *PRL* gene single-nucleotide polymorphism (SNP) with SCZ.^74^

Studies of *DR2D* common variants show unclear results in T2D.^67, 75, 76^ Recent human studies report that the PRL regulatory element (PREB) is associated with eating disorders,^77^ and that a variant near the PRL gene (*PRL*) is associated with obesity in males.^78^ A *PRL* gene SNP shows significant associations with lower glycemia during a 2-h oral glucose tolerance tests and a higher level of beta-cell function in a Chinese study.^79^

## Dopamine and PRL pathway hypothesis for the SCZ–T2D comorbidity: needed studies

It is noteworthy that both low as well as high levels of a hormone may cause a pathological state. Furthermore, the basis for decreased or increased hormonal levels may remain within constitutional hormonal secretion, as well as within hormonal receptor affinity, which both may be determined by genetic variations.

As PRL is either decreased or increased in SCZ drug-naive patients, functional impairment and disease pathogenesis may both depend on reduced as well as increased hormone level or action. Similarly, dopamine pathway alterations may lead to the psychotic symptoms of SCZ.

As in SCZ,^36, 37^ T2D can as well be caused by both decreased levels of PRL as well as by PRLR dysfunction leading to increased PRL levels. Higher PRL levels in drug-naive SCZ subjects may be due to PRLR resistance, which may lead to reduced PRL action in brain areas important for social and cognitive functions, as well as to impaired beta-cell insulin secretion and insulin sensitivity, the latter being correlated to increased PRL levels in older adults compared with young adults.^52^

**Figure 1:**
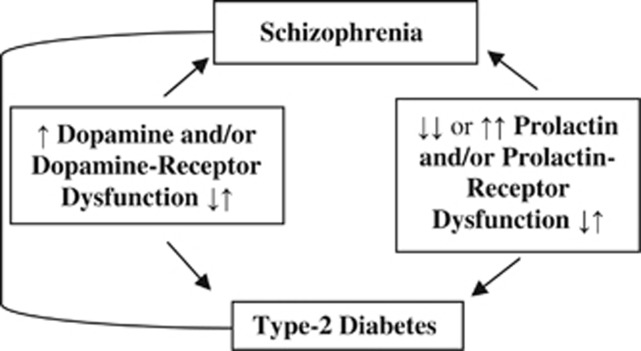
Hypothesized relationship between increased dopamine and/or dopamine receptor dysfunction (increased or decreased affinity), decreased or increased prolactin and/or prolactin-receptor dysfunction (increased or decreased affinity), and resultant schizophrenia and type 2 diabetes.

We postulate that some subjects with PRL pathway dysfunction, resulting in either lower PRL levels due to dopamine receptor hyperfunction, or higher PRL levels due to DR2D dysfunction or PRLR resistance, may sustain disrupted mental development (Figure 1).

We hypothesize that the *PRL* and/or *PRLR* gene may carry risk variants associated with T2D-correlated metabolic/psychiatric traits, and contribute to SCZ, T2D and/or to their clinical association.

Our hypothesis is innovative as, to our knowledge, to date there are no data in the literature regarding genetic screening of whole *PRL* and *PRLR* genes in T2D and/or SCZ, except for one *PRL* SNP association to SCZ.^74^

Dopamine action is mediated by the dopamine receptors DR1D, DR2D, DR3D, DR4D and DR5D. Both increased dopamine function in certain brain areas and decreased dopamine action in others may contribute to SCZ as well as to T2D. Consequently, we postulate that dopamine receptor gene variants may confer risk for the comorbidity of SCZ and T2D, as well as for only SCZ or T2D.

Worth mentioning, the *DR1D* gene is on 5q35.1, a locus linked to SCZ^80^ and C-reactive protein in T2D families;^81^ the *DR2D* gene is on 11q23, a locus linked to both T2D and SCZ.^82, 83^ Furthermore, the *PRL* gene on 6p22.2-21.3, the *DR3D* gene on 3q13.3, the *DR4D* gene on 11p15.5 and the *DR5D* gene on 4p16.1 are located in loci showing common genetic risk inheritance for both T2D and SCZ.^8^ The *PRLR* on 5p13.2 lies in a locus strongly linked to SCZ^84^ and nearby the locus linked to C-reactive protein in T2D families.^81^ For all the above-mentioned reasons, we believe that the genes underlying the dopamine and PRL pathways are of high interest to the genetics of SCZ–T2D comorbidity. It would be thus important to screen the *PRL*, *PRLR* and dopamine receptor *DR1D*, *DR2D*, *DR3D*, *DR4D* and *DR5D* genes in both T2D and SCZ families and case–control groups.

It would be important to perform linkage and association tests not only in a group of patients with T2D or SCZ but also with associated traits and pre-phenotypes leading to both diseases, as a powerful innovative strategy for gene identification. This suggested method stems from the hypothesis that T2D is a complex disorder potentially characterized by abnormal neuroendocrine and psychological traits, and consequential abnormal behavioral compensatory mechanisms.

These combined neuroendocrine–psychological–behavioral traits may, potentially in the setting of risk susceptibility to slow metabolic activity and beta-cell dysfunction, allow in the long term to develop T2D. Similarly, analogous abnormal mental traits, which if untreated will lead to SCZ, may contribute, in the setting of predisposition to beta-cell failure, to the development of pre-diabetes and T2D. In addition, we predict that complex gene variants as well as unique gene mutations present in major and minor traits of disease may lead to the onset of the full-complex disorders.^85^ We postulate that the SCZ–T2D association may mask common pathological pathways, and that either the impaired dopamine and/or PRL pathway in SCZ may impair glucose homeostasis, thereby contributing to T2D. However, certain gene variants may explain both SCZ and T2D, some variants may influence only T2D and others may be unique to SCZ.

### Pathway interplay and expected molecular stratification

Despite dopamine pathway's role in SCZ being accepted, it is uncertain to what extent it contributes to disease, and certainly other environmental factors and pathways have a role as well in the disease pathogenesis.^86^ Most likely, SCZ subjects will not all share an identical pathogenesis; heterogeneity of the clinical manifestations will potentially correspond to molecular heterogeneity.

Among the possible SCZ pathways, the serotonergic pathways may have a role in T2D as well.^87, 88^ Inhibition of peripheral serotonin synthesis reduces obesity and metabolic dysfunction,^87^ while increased expression of 5-HT_2C_ receptors in pancreatic beta cells may inhibit insulin secretion.^88^ The dopaminergic and serotoninergic pathway interact; mutant mice over-expressing DR2D receptors in the striatum, exhibit both decreased willingness to work for reward, as well as upregulation of 5-HT_2C_ receptors, which may lead to impaired insulin secretion.^89^

These data anticipate the complex heterogeneity of the molecular genetics underlying the different SCZ subtypes and manifestations. It is unlikely that a single pathway or few genes in a specific pathway will explain either SCZ, or T2D, or their clinical association. Genetic stratification in disease predisposition correlated to phenotype heterogeneity is expected.

As not all SCZ patients develop T2D, and only some T2D subjects will present psychological traits overlapping with pre-symptoms of SCZ, we hypothesize that a subgroup of individuals comorbid for both SCZ and T2D may have inherited genetically impaired dopamine and PRL pathways. It would be necessary to stratify the genetic risk in these pathways, and correlate it with sub-phenotypes of SCZ and T2D. The correlation of the genes and their variations with the phenotype(s), or the lack of thereof, may further elucidate the genetic basis of the SCZ–T2D association.

### Possible impact

Our proposed theory challenges the currently accepted paradigm that T2D is only a metabolic disorder, and that SCZ is merely a mental disorder, which affects few unlucky individuals carrying unfavorable gene variants. Our hypothesis may produce a shift in the attention given to subjects with T2D-familial risk in terms of psychological traits contributing to disease. In subjects with family history for SCZ, our hypothesis may contribute to major awareness toward the metabolic phenotypes of pre-diabetes. Thus, the creation of a new way to think about the mental alterations in SCZ and the metabolic dysfunction of T2D will open a new horizon of approaches, experimental designs and, in the long term, possibly cognitive–behavioral and preventive therapies for at-risk subjects. In fact, individuals with SCZ often have a gradual period of decline over 1–2 years before development of overt psychosis, referred to as ‘prodromal' or ‘ultrahigh-risk state',^90^ so there is a window of opportunity for early therapeutic interventions. If our theory is proven valid via genetic studies determining the gene variants in the dopamine–PRL pathway predisposing to SCZ and consequently to T2D, screening for disease biomarkers, such as PRL levels, or for gene risk-variant carriers could be performed in children and teenagers with increased SCZ-familial risk or presenting with cognitive, developmental, emotional and/or behavioral issues, resembling possible predisposition to SCZ. Finally, if these young subjects were confirmed to carry a genetic risk variant for SCZ, they could be evaluated and monitored more closely regarding their mental and cognitive well-being in their common environment, and stress factors prevention and reduction at school, in the family and in the peer setting could be implemented. Similarly, targeted nutrition and exercise programs, as well as mind–body calming biofeedback techniques could be realized. To prove our idea, different patient sub-phenotypes per disorder would be required to identify a potential common neuro–endocrine–mental–metabolic dysfunction. By proving that gene variants responsible for one disorder are contributing to the other disorder, or to any of its associated traits, the foundation of the genetic overlap between the two diseases could be built, as well as of the existence of new distinct genetically combined metabolic and mental–behavioral traits.

## Beyond our hypothesis

### Pleiotropic theory, causality theory, disease model

We believe in the hypothesis of the existence of some risk genes with independent pleiotropic effects, consequently causing both T2D and SCZ. One example may be represented by the *PSMD9* gene, which we show as strongly linked to T2D,^91^ and others report as one of the major gene players in SCZ.^92^ PSMD9 is a co-activator and interacting partner of several proteins, thus it may well have pleiotropic effects on different pathways, as well as mediate inflammation.^93^

However, we consider the causality hypothesis as having stronger impact on the SCZ–T2D comorbidity. In fact, an impaired pathway in early-onset SCZ may determine T2D over time.

Of note, some patients with both diseases may have genes involved independently in each disease as well, and not sharing the common pathway of SCZ–T2D or only partially responsible for it.

We also need to consider that the SCZ–T2D comorbidity may be due (1) an oligogenic disease model for some families with mildly deleterious mutations in few genes, as well as (2) a monogenic or digenic disease model for other families with frankly deleterious mutations in one or two genes, respectively, and finally (3) a polygenic disease model where the contribution of several genes with modestly deleterious mutations is needed for the diseases to manifest.

### Other candidate genes for SCZ–T2D comorbidity

There are several candidate genes for SCZ–T2D comorbidity and they mostly derive from the overlapping data in the T2D and SCZ genome-wide association studies.^8^ We hereby report some of the identified candidate genes for both SCZ and T2D, and describe our view on their potential limitations for the SCZ–T2D comorbidity, especially for a possible monogenic, digenic or oligogenic disease model, in which candidate genes specifically affecting SCZ and T2D common pathways are needed to establish comorbidity.

Superoxide dismutase 2 (*SOD2*) is associated with diabetic complications^94^ and T2D,^95^ but not all studies confirm an association with T2D.^96^ No data confirm an association with SCZ. Glutathione-*S-*transferase M1 (*GSM1*) is associated with T2D^97^ and SCZ.^98^ As oxidative stress is present in aging disorders of the general population at a higher prevalence than the SCZ–T2D association, we do not think that oxidative stress is the cause of SCZ–T2D comorbidity, but that a *GSM1* or *SOD2* variation may predispose to inability to counter-effect oxidative stress triggered by other factors. Apolipoprotein D (*APOD*) is associated to T2D,^99^ but its implicated role in SCZ is not confirmed in all studies.^100^ Wolfram syndrome 1 (*WFS1*) confers risk to T2D,^101^ and, despite high mental disorder prevalence (but not SCZ) in patients with Wolfram syndrome, *WFS1* role in SCZ is not supported.^102, 103^

Despite impairment of epidermal growth factor (EGF) in SCZ-autoptic brains^104^ and islet cells of T2D patients,^105^ *EGF* association studies with SCZ are inconsistent,^106, 107^ and to our knowledge, there are currently no genetic studies of *EGF* in T2D.

Tumor necrosis factor (TNF) is a cytokine with effects on insulin resistance, but data showing a genetic role for *TNF* in T2D are inconsistent.^108, 109^ TNF contribution is reported in SCZ.^110^ Data show that interleukin-6 and interleukin-1 beta may contribute to the SCZ–T2D link.^8^ Even though inflammatory molecules such as TNF, interleukin-6 and interleukin-1 beta may contribute to both disease pathogenesis, and inherited gene variants of these inflammatory molecules may predispose to increased inflammation, the inflammation-driven pathogenic role is likely not the prior one contributing to SCZ–T2D comorbidity, as inflammation is present in aging disorders at a higher prevalence than the SCZ–T2D association.

Methylenetetrahydrofolate reductase contributes to homocysteine metabolism and hyperhomocysteinemia is associated with T2D^111^ and SCZ,^112^ but it may represent a disorder epiphenomenon.

The strongest T2D-risk gene, *TCF7L2*, is associated with SCZ in few studies;^113, 114^ however, *TCF7L2* odds ratio for both diseases does not explain the genetic predisposition risk (T2D *λ*_s_≈3 and SCZ *λ*_s_≈2).

Of note, studies have failed to replicate findings of other common SNPs significantly associated with T2D and psychotic disorders.^115^ However, the variable allele frequencies and linkage disequilibrium blocks among different ethnic groups and the sample sizes used are limitations of these studies. Most importantly, the SNPs significantly associated in genome-wide association studies may only be a marker of the causative disorders variants, thus explaining the difficulty in replication.

There is no consensus on androgen receptor's (*AR*) role in SCZ;^116, 117, 118^ *AR* variants are associated with obesity in T2D men,^119^ and androgens may be implicated in beta-cell dysfunction.^120^ Thus, *AR* gene may confer risk for both T2D and SCZ, and its role in SCZ–T2D comorbidity cannot be ruled out.

Neuropeptide Y (*NPY*) gene is associated with T2D^121^ and SCZ,^122^ but less consistently with SCZ.^123^ NPY is of interest to the hypothesized correlation of the dopamine–PRL pathway with the SCZ–T2D comorbidity, as NPY appears, beyond stimulating appetite, to amplify the inhibitory action of dopamine on PRL secretion.^124^ Thus, an altered NPY pathway may have effects on the dopamine–PRL pathway. It is our understanding that the NPY pathway represents, together with the serotonergic pathway, another possible player in the SCZ–T2D association.

### Epigenetics, SCZ and T2D

There is evidence that epigenetic pathways interacting with genetic and environmental factors have a role in the pathogenesis of T2D and SCZ. Early-life environmental factors, gestational and birth abnormalities (for example, low birth weight) and epigenetic programming emerge as risk factors for T2D^125^ and SCZ/psychotic disorders,^126^ indicating that phenomena of adaptation may contribute to these diseases. Early growth and prenatal abnormalities predict in the long term—via epigenetic pathways—an increased risk for T2D.^127^ One of many examples of possible interplay between genetics and epigenetics in diabetes is given by the maturity-onset diabetes of the young 4 (MODY4) *IPF-1* gene, an essential transcription factor in pancreas development and proliferation. While the MODY4 *IPF-1 P33T* variant contributes to monogenic diabetes, gestational diabetes, low birth weight, miscarriages and early postnatal death,^128^ fetal undernutrition silences the transcription of Pdx1 (rat homologous of MODY4 *IPF-1*) via promoter DNA methylation and histone modifications.^129^

In addition, below-average-sized babies carry a significantly higher risk for SCZ spectrum disorders.^130^ Hormones affect adult metabolism and epigenetic programming,^131^ which may affect dopamine, *NMDA* and *GABA* gene function contributing to SCZ.^132^ Moreover, *DRD3* gene hypermethylation is significantly associated with SCZ risk.^133^ Of great interest, DNA methylation of the *DRD2* regulatory region is described in monozygotic twin pairs, one concordant and one discordant for SCZ; the affected twin from the pair discordant for SCZ is epigenetically closer to the affected concordant twins than to his unaffected monozygotic co-twin.^134^ In drug-naive SCZ patients, serotonin transporter (*5-HTT*)-reduced expression is correlated with *5-HTT* promoter DNA hypermethylation, indicating that *5-HTT* epigenetic hypo-activity is linked to SCZ.^135^ The same author also reports epigenetic alterations of the dopaminergic system in SCZ and bipolar disorder.^136^ In SCZ, the soluble catechol-*O*-methyltransferase (*S-COMT*) gene is identified as significantly hypermethylated as well as a dysregulated epigenome, the latter more pronounced in early-onset patients.^137^ There are currently no studies of PRL pathway epigenetics in T2D or SCZ.

## Conclusion

We strongly advocate new research in the area of associated mental and metabolic disorders to create a new focus on the neuroendocrine–mental–metabolic dysfunctions, which may characterize pre-disease states, and develop novel prevention strategies that address both conditions. If we open our eyes to the idea of this joint pathogenesis of, at first sight, so different disorders, we might be able to advance research in early prevention and treatment for both conditions.

In summary, there is a scientific need for the development of a new focus on the genetic predisposition to shared mental and metabolic dysfunctions, gender-related different genetic susceptibility, epigenetic mechanisms and their long-term effects on mental illness and T2D. As a result, new genetic research, experimental studies and longitudinal investigations are now needed. In the long term, clinical trials might be started: new therapies such as nutritional preventions (for example, diet with controlled or reduced tyrosine intake) may prevent or counteract dopamine pathway impairment in subjects at risk for increased dopamine levels or dopamine receptor function before both their first-episode psychosis onset and T2D. In addition, later on, early environmental and pharmacological interventions may reduce the prevalence of both SCZ and T2D. Furthermore, therapies with PRL agonists acting on the beta cell, insulin action and brain may reduce T2D risk and SCZ-related symptoms. We do expect that within SCZ and T2D there might be a subgroup of patients with genetic, epigenetic and functional impairment of the dopamine–PRL pathway, and that this line of investigation will potentially lead to the application of precision medicine principles to T2D and SCZ, potentially even before the first episode of the psychotic disorder, and perhaps even before its prodrome became clinically apparent.
